# Letter to the Editor in Reference to “New Onset Vertigo After COVID-19 Infection” – COVID-19-related Vestibular Neuritis: Case Series and Review of the Literature

**DOI:** 10.1007/s12070-022-03201-2

**Published:** 2022-11-16

**Authors:** Andrea Frosolini, Daniela Parrino, Cristoforo Fabbris, Giacomo Spinato, Cosimo de Filippis

**Affiliations:** 1 Department of Medicine - DIMED, University of Padua, Treviso, Italy; 2 Department of Neurosciences, University of Padua, Treviso, Italy; 3 Unit of Otolaryngology, Treviso Hospital, AULSS 2, Treviso, Italy; 4 Unit of Audiology, Treviso Hospital, AULSS 2, Treviso, Italy; 5 Unit of Otolaryngology and Regional Centre for Head and Neck Cancer, Treviso Hospital, AULSS 2, Treviso, Italy

**Keywords:** SARS-CoV-2, COVID-19, Audiovestibular disorders, Vertigo, Dizziness, Audiovestibular evaluation

## Dear Editor,

We read with interest the article by Motawea et al. reporting the case of a sudden vertigo in a 60-year-old woman with a confirmed Coronavirus-Disease-19 (COVID-19) [1]. The authors concluded that Severe-Acute-Respiratory-Syndrome-Coronavirus-2 (SARS-COV-2) may lead to vestibular neuritis (VN), but more well-designed observational studies with a larger sample size are needed to establish a definite association between COVID-19 and vertigo.

The incidence of vestibular disorders in COVID-19 patients is still unknown and varies across different studies. We reviewed the international literature of COVID-19-related vestibular disorders and the electronic database at our department, a tertiary referral centre for audiovestibular disorders. In January 2022 a structured search of the English literature published on PubMed was performed by searching the terms “vestibular neuritis” and “COVID-19”. Only inherent reports with SARS-CoV-2-positive patients, as confirmed by molecular nasopharyngeal swab, and with detailed clinical and diagnostic data were considered. Overall, 15 papers were retrieved and 6 were included (Table 1).

**Table 1:** Clinical data, evaluation, diagnosis treatment and outcome of the patients from literature review on vestibular neuritis and COVID-19, including the present cases report

1st Author (year of publication)	Sex, age	Comorbidity and any AV past history	COVID-19 classification - duration - symptoms	AV Symptoms, - Days from COVID-19 positivity to onset	Evaluation	Vestibular signs	Diagnosis	Therapy	Follow-up and outcome
Mat (2021)^1^	F, 13	NR	Mild - NR - NR	Rotatory vertigo, vomiting − 0	VNG; vHIT; ENT and neurological evaluation; audiometry	Right spontaneous Ny; left deviation (Fukuda test)	Left COVID-19-induced vestibular neuritis	VR	1 month, symptom resolution
Vanaparthy (2020)^2^	F, 63	Aplastic anemia, mitral valve prolapse, celiac disease. Motion sickness	Mild − 2 month - GS, facial spasm, anosmia, disgeusia, skin rash, Raynaud’s phenomenon	Rotatory vertigo, vomiting, unsteady gait − 65	VNG, ENT and neurological evaluation	Right spontaneous Ny	Left COVID-19-induced vestibular neuritis	60 mg oral prednisone, 10 days tapered; VR	NR
Malayala (2021)^3^ Case 1	F, 31	NR	Mild - NR - GS	Rotatory vertigo, unsteady gait − 13	MRI brain; ENT and neurological evaluation; audiometry	NR	COVID-19-induced vestibular neuritis	60 mg oral prednisone, 10 days tapered; VR	1 month, symptom resolution
Malayala (2021)^3^ Case 2	F, 29	NR	Moderate - NR -NR	Rotatory vertigo, vomiting − 0	CT chest; CT cerebral; MRI brain; ENT and neurological evaluation; audiometry	NR	COVID-19-induced vestibular neuritis	Intravenous steroids (DNS); VR	NR
Giannantonio (2021)^4^	M, 13	NR	Mild - NR - GS	Rotatory vertigo, vomiting, unsteady gait − 0	MRI brain; ENT evaluation; audiometry	Right spontaneous Ny, positive HIT	Left COVID-19-induced vestibular neuritis	20 mg intravenous prednisone, 10 days tapered	1 month, symptom resolution
Aasfara (2021)^5^	F, 36	Pregnancy (37 weeks)	Mild - NR - NR	Rotatory vertigo, vomiting, right ear hipoacusia − 42	MRI brain, VNG with Caloric test, ENT evaluation, audiometry, electromyography, lumbar puncture	Left spontaneous Ny, Hyporeflexia	Right COVID-19-induced cochlear-vestibulopathy and facial palsy	Intravenous steroids (DNS); VR	1 month, symptom resolution
Frosolini (present letter)	F, 31	None	Mild − 1 month - GS anosmia, dysgeusia	Rotatory vertigo, vomiting, unsteady gait − 2	VNG with Caloric test, ENT evaluation, audiometry	Hyporeflexia	Left COVID-19-induced vestibular neuritis	VR	1 month, symptom resolution
Frosolini (present letter)	M, 44	None	Mild − 9 days - GS anosmia, dysgeusia	Rotatory vertigo, vomiting, unsteady gait − 32	VNG with Caloric test, ENT evaluation, brain CT, audiometry	Left spontaneous Ny, positive HIT	Right COVID-19-induced vestibular neuritis	50 mg oral prednisone, 10 days tapered; VR	1 month, symptom resolution

Abbreviations: Audiovestibular (AV), Coronavirus Disease 19 (COVID-19), Computed Tomography (CT), Drug Not Specified (DNS) Ear Nose and Throat (ENT), General symptoms like fever, fatigue, miastenia, and cough (GS), Head Impulse Test (HIT), Magnetic Resonance Imaging (MRI), Not Reported (NR), Nystagmus (Ny), video Head Impulse Test (vHIT), Videonystagmography (VNG), Vestibular Rehabilitation

^1^ Mat Q, Noël A, Loiselet L, Tainmont S, Chiesa-Estomba CM, Lechien JR et al. (2021) Vestibular Neuritis as Clinical Presentation of COVID-19. Ear Nose Throat J. 11:145561321995021. doi: 10.1177/0145561321995021. Epub ahead of print. PMID: 33,570,425

^2^ Vanaparthy R, Malayala SV, Balla M (2020) COVID-19-Induced Vestibular Neuritis, Hemi-Facial Spasms and Raynaud’s Phenomenon: A Case Report. Cureus 12, e11752

^3^ Malayala SV, Mohan G, Vasireddy D, Atluri P (2021) A case series of vestibular symptoms in positive or suspected COVID-19 patients. Infez Med 29, 117–122

^4^ Giannantonio S, Scorpecci A, Montemurri B, Marsella P (2021) Case of COVID-19-induced vestibular neuritis in a child. BMJ Case Rep. 1;14(6):e242978. doi: 10.1136/bcr-2021-242978. PMID: 34,078,625; PMCID: PMC8173285

^5^ Aasfara J, Hajjij A, Bensouda H, Ouhabi H, Benariba F (2021) A unique association of bifacial weakness, paresthesia and vestibulocochlear neuritis as post-COVID-19 manifestation in pregnant women: a case report. Pan Afr Med J 13;38:30. doi: 10.11604/pamj.2021.38.30.27646. PMID: 33,777,298; PMCID: PMC7955605.

In 5 French hospitals, over the period February-May 2020, comparing with 2018 and 2019, no significant increase in admission for acute peripheral vestibulopathy (APV) was observed. Moreover, significant differences among hospitals located in COVID-19 high- and low-risk zones, or significant increase in the severity of the APV cases was observed. Accordingly, a retrospective review of acute cochleovestibular disorders after and before pandemic conducted at our department found no significant changes regarding incidence of APV.[2].

Only 6 cases of instrumentally confirmed VN simultaneous to COVID-19 have been reported to date, and two more cases occurred at our department. Differential diagnosis has to be considered regarding a first episode of Ménière’s disease or vestibular migraine attack, thus an accurate diagnostic workout should be mandatory.[3] The mechanism by which SARS-CoV-2 can cause VN is unclear and speculative. Motawea et al. supported viral and post-viral inflammatory disorders.[1] Indeed, the cell receptor angiotensin-converting enzyme 2 (ACE2), which allows intracellular entry of SARS-CoV-2, has been found in nasal tissues in murine and human model and in Eustachian tube, middle ear and cochlear tissues in murine model. On the other hand, physical and emotional stress experienced by infected people could play a role in the expression of vestibular symptoms – as occurred for headache patients – or could have triggered the reactivation of possible latent viruses (e.g., HSV-1). The latter mechanism could be hypothesised especially in patients in which VN was not present at COVID-19 symptoms onset but later during the course of the disease.[4, 5].

To the best of our knowledge, even if COVID-19 infection seems not to increase the risk of VN occurrence, it would be appropriate to routinely test for SARS-CoV-2 infection patients with diagnosed VN. Moreover, prospective studies on a large series of COVID-19 patients should try to better define the epidemiology of cochlear-vestibular involvement and elucidate the SARS-CoV-2-related prognosis on peripheral and central audiovestibular functions. Among patients that can’t be easily visited due to quarantine regimen, a telemedicine evaluation could be helpful.
